# Prevalence and clinical predictors of spasticity after intracerebral hemorrhage

**DOI:** 10.1002/brb3.2906

**Published:** 2023-02-07

**Authors:** Ling‐Yi Liao, Pei‐Dong Xu, Xiang‐Qin Fang, Qing‐Hua Wang, Yong Tao, Huan Cheng, Chang‐Yue Gao

**Affiliations:** ^1^ Department of Rehabilitation Medicine, Daping Hospital Army Medical University Chongqing China; ^2^ Department of Information, Daping Hospital Army Medical University Chongqing China

**Keywords:** cerebral hemorrhage, muscle spasticity, rehabilitation, stroke

## Abstract

**Background:**

Spasticity is a common complication of intracerebral hemorrhage (ICH). However, no consensus exists on the relation between spasticity and initial clinical findings after ICH.

**Methods:**

This retrospective study enrolled adult patients with a history of ICH between January 2012 and October 2020. The modified Ashworth scale was used to assess spasticity. A trained image analyst traced all ICH lesions. Multivariable logistic regression was used to examine the association between ICH lesion sites and spasticity.

**Results:**

We finally analyzed 304 patients (mean age 54.86 ± 12.93 years; 72.04% men). The incidence of spasticity in patients with ICH was 30.92%. Higher National Institutes of Health stroke scale (NIHSS) scores were associated with an increased predicted probability for spasticity (odds ratio, OR = 1.153 [95% confidence interval, CI 1.093–1.216], *p* < .001). Logistic regression analysis revealed that lower age, higher NIHSS scores, and drinking were associated with an increased risk of moderate‐to‐severe spasticity (OR = 0.965 [95% CI 0.939–0.992], *p* = .013; OR = 1.068 [95% CI 1.008–1.130], *p* = .025; OR = 4.809 [95% CI 1.671–13.840], *p* = .004, respectively). However, smoking and ICH in the thalamus were associated with a reduced risk of moderate‐to‐severe spasticity (OR = 0.200 [95% CI 0.071–0.563], *p* = .002; OR = 0.405 [95% CI 0.140–1.174], *p* = .046, respectively) compared with ICH in the basal ganglia.

**Conclusions:**

Our results suggest that ICH lesion locations are at least partly associated with post‐stroke spasticity rather than the latter simply being a physiological reaction to ICH itself. The predictors for spasticity after ICH were age, NIHSS scores, past medical history, and ICH lesion sites.

## Graphical abstract

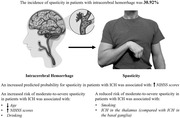

## 1 INTRODUCTION

Spasticity is a common complication of stroke with a pooled incidence of 25.3% (Nam et al., 2019; Zeng et al., 2020). A velocity‐dependent increase in resistance during passive stretching is a characteristic of spasticity, which is due to the hyperexcitability of the stretch reflex (Li & Francisco, 2015). Muscle spasticity as a result of cerebral hemisphere injury can lead to long‐term disability (Li, 2020; Zeng et al., 2020). The onset of spasticity is highly variable and may occur shortly or more than 1 year after stroke (Ward, 2012). Katoozian et al. (2018) identified a 2.5 times higher risk of spasticity in patients with hemorrhagic stroke than in patients with ischemic stroke during the 3 months post‐stroke. A recent meta‐analysis also identified hemorrhagic stroke as a risk factor for post‐stroke spasticity (odds ratio, OR = 1.879 [95% confidence interval, CI 1.418−2.490], I2 = 27.3%) (Zeng et al., 2020). However, the role of hemorrhagic stroke in predicting post‐stroke spasticity requires further exploration. Moreover, hemorrhagic stroke may not be an independent risk factor for post‐stroke spasticity, and damage to the basal ganglia region may lead to greater spasticity in patients with hemorrhagic stroke (Jin & Zhao, 2018; Zeng et al., 2020). It remains uncertain whether the risk relates to the location of the intracerebral hemorrhage (ICH) or indicates a reaction to the ICH lesion itself. Although BoNT‐A is an established treatment for focal spasticity (Francisco et al., 2021), advanced well‐defined spasticity management strategies have not been conclusively established (Mahmood et al., 2019; Salazar et al., 2019). Patients with spasticity who have low motor recovery potential may benefit from aggressive treatment and care (Li, 2017); therefore, clarifying an association between post‐ICH spasticity and ICH lesion location would be beneficial for correctly following up high‐risk patients and effectively guiding resources (Zeng et al., 2020). Herein, we hypothesized that the incidence and degree of post‐stroke spasticity would differ among ICH lesion sites, with the highest incidence in patients with lesions in the basal ganglia region. Moreover, despite its high incidence, no consensus exists (Nam et al., 2019) on the number of patients who develop spasticity after ICH and (Zeng et al., 2020) the relation between spasticity and initial clinical findings after ICH. Therefore, this study aimed to investigate the prevalence of spasticity after ICH and identify clinical predictors of subsequent spasticity.

## 2 MATERIALS AND METHODS

### 2.1 Study population

This study was approved by the Biomedical Ethics Committee of the Army Medical Center of the People's Liberation Army. We enrolled consecutive patients with ICH confirmed by computed tomography or magnetic resonance imaging (MRI) between January 2012 and October 2020 in a prospective cohort study. Patients with subarachnoid hemorrhage were excluded. Patient characteristics were prospectively recorded, including age, sex, education, insurance, past medical history, lesion sites, lesion side, and National Institute of Health stroke scale (NIHSS) scores after acute intervention.

### 2.2 Lesion tracing

Compared with the relative contralateral brain region, lesions were visually identified as having altered fluid‐attenuated inversion recovery (FLAIR) and diffusion‐weighted imaging (DWI) signal intensities. We used DWI sequences for MRI within 48 h post‐stroke and FLAIR sequences for MRI within 48 h to 7 days post‐stroke (Picelli et al., 2014). A trained image analyst traced all lesions, and an experienced clinical neurologist confirmed them. Both were blinded to all clinical data, except for the side of the hemiparesis.

### 2.3 Spasticity measure

Spasticity assessments of the upper/lower extremities and trunk were performed using the modified Ashworth scale (MAS), the most widely used clinical scale for assessing increase in muscle tone, which manifested as increased resistance of joints to passive movement on a scale ranging from grade 0 to grade 4 (non‐spastic: MAS 0; mild: MAS 1, 1+; moderate: MAS 2, 3; severe: MAS 4) (Meseguer‐Henarejos et al., 2018). Grade 0 denoted normal or lowered muscle tone. Any spasticity was defined as an MAS score ≥1 for any of the passive movements performed. For MAS, higher grades equated to more severe spasticity. The MAS is considered to provide reliable and reproducible results (Bohannon & Smith, 1987). The assessments were done by a trained physiotherapist at a 1‐year follow‐up. Patients with MAS scores ≥1 in any one joint were considered to have spasticity.

### 2.4 Statistical analysis

All variables in the study were calculated using descriptive statistics. The *χ* ^2^ test or Fisher's exact test was used to compare the categorical variables. The *t*‐test or Wilcoxon rank‐sum test was used to calculate continuous variables. Multivariable logistic regression was used to calculate the association between the ICH lesion sites and MAS scores. Differences were considered statistically significant at a *p*‐value of below .05. Data management and analyses were performed using SPSS (version 26.0; IBM Corp., Armonk, NY, USA). This study conformed to STROBE guidelines. The STROBE checklist is included in the Supporting Information.

## 3 RESULTS

In total, 304 patients with ICH (mean age 54.86 ± 12.93 years; 72.04% men) were eligible for the study. The patient characteristics are shown in S1. Among the 304 patients, 94 experienced spasticity (31%). The results indicated that patients with spasticity were younger than those without spasticity (*p* = .014; Table S1). NIHSS scores were higher in ICH patients with spasticity (*p* < .001; Table S1).

The spasticity characteristics at different lesion locations are shown in Table 1. It is apparent from the table that no patient with ICH in the cerebellum or other regions had spasticity. The incidence of spasticity in patients with brainstem hemorrhage was the highest (36.84%) among all the lesion locations. However, from these data, we can see that patients with ICH in the basal ganglia had the highest incidence of moderate‐to‐severe spasticity (24.02%).

**TABLE 1:** The characteristics of spasticity in different lesion locations.

Lesion site	Spasticity *n* (column %)	Degree of spasticity			
		Non‐spastic	Mild	Moderate	Severe
Lobes of brain	16 (36.36%)	28 (63.64%)	10 (22.73%)	6 (13.64%)	0
Thalamus	7 (28.00%)	18 (72.00%)	4 (16.00%)	3 (12.00%)	0
Basal ganglia	59 (32.96%)	120 (67.04%)	16 (8.94%)	35 (19.55%)	8 (4.47%)
Cerebellum	0	3 (100.00%)	0	0	0
Brainstem	7 (36.84%)	12 (63.16%)	4 (21.05%)	2 (10.53%)	1 (5.26%)
Multiple sites	5 (17.24%)	24 (82.76%)	4 (13.79%)	1 (3.45%)	0
Else	0	5 (100.00%)	0	0	0

*Note*: Values are reported as *n* (column %). All lesions are isolated to these regions except multiple sites.

The multivariable logistic regression analysis for the potential predictor variables for spasticity is presented in Table 2. NIHSS scores were significant for predicting the occurrence of spasticity. Higher NIHSS scores were associated with an increased probability of spasticity (OR = 1.153, *p* < .001). Similarly, the results revealed that higher NIHSS scores and younger age were associated with moderate‐to‐severe spasticity compared with mild spasticity (OR = 1.068, *p* = .025; OR = 0.965, *p* = .013, respectively). Smoking could reduce the risk of moderate‐to‐severe spasticity (OR = 0.200, *p* = .002). Conversely, drinking was associated with a higher risk of moderate‐to‐severe spasticity (OR = 4.809, *p* = .004). However, no significant differences were found between the occurrence of spasticity and the different ICH lesion locations. However, further analysis showed that patients with ICH in the thalamus or multiple sites had a lower risk of moderate‐to‐severe spasticity (OR = 0.405, *p* = .046; OR = 0.094, *p* = .026, respectively) than patients with ICH in the basal ganglia.

**TABLE 2:** Multivariable logistic regression analysis for the prediction of any spasticity and moderate‐to‐severe spasticity

Predictors	Wald	OR	*p*‐Value	95% CI	Wald	OR	*p*‐Value	95% CI
	Any spasticity				Moderate‐to‐severe spasticity			
Age	3.082	0.979	0.082	0.956–1.003	6.215	0.965	**0.013**	0.939–0.992
Gender	0.002	1.016	0.963	0.509–2.028	0.687	1.418	0.407	0.621–3.235
NIHSS	27.369	1.153	**<0.001**	1.093–1.216	5.011	1.068	**0.025**	1.008–1.130
Hypertension	0.000	0.995	0.990	0.433–2.288	1.631	1.841	0.202	0.722–4.697
Diabetes	0.002	0.983	0.962	0.475–2.034	0.308	1.283	0.579	0.533–3.089
Hyperlipidemia	0.643	0.748	0.423	0.367–1.522	0.006	0.968	0.939	0.416–2.254
Coronary	0.003	1.029	0.957	0.364–2.908	3.448	0.340	0.063	0.109–1.062
Smoking	0.377	0.765	0.539	0.325–1.799	9.299	0.200	**0.002**	0.071–0.563
Drinking	0.695	1.422	0.404	0.610–3.407	8.479	4.809	**0.004**	1.671–13.840
Volume	3.749	0.572	0.053	0.324–1.007	0.238	0.847	0.625	0.436–1.647
Lesion sites (compared with basal ganglia)								
Lobes of brain	0.469	1.326	0.494	0.591–2.975	0.101	0.803	0.750	0.207–3.106
Thalamus	0.307	1.338	0.580	0.477–3.750	2.771	0.405	**0.046**	0.140–1.174
Brainstem	0.212	1.288	0.645	0.440–3.772	0.834	0.538	0.361	0.143–2.033
Multiple sites (≥2)	2.349	0.418	0.125	0.137–1.275	4.941	0.094	**0.026**	0.012–0.756

*Note*: Bold values indicate *p* < .05.

Abbreviations: CI, confidence interval; NIHSS, the National Institute of Health stroke scale; OR, odds ratio.

## 4 DISCUSSION

This analysis of patients with ICH found that the occurrence of post‐stroke spasticity was associated with NIHSS scores, and the degree of spasticity was related to age, NIHSS scores, smoking, drinking, and lesion location. Our findings are consistent with those of previous studies showing that NIHSS scores are significant clinical predictors of post‐stroke spasticity (Glaess‐Leistner et al., 2021; Schinwelski et al., 2019). Moreover, predictors for the development of spasticity after ICH were age, NIHSS scores, past medical history, and ICH lesion sites.

The relationship between age and spasticity has never been specifically explored in stroke, although it has been identified as an important predictor of stroke severity, risk factors, and complications (Lundstrom et al., 2008; Shin et al., 2018; Tedesco Triccas et al., 2019). Our analysis showed that moderate‐to‐severe post‐stroke spasticity is more likely to occur in the younger population, which is consistent with the results of Lundström et al. (2008). However, several studies have reported an association between increasing age and risk for increasing muscle tone (Persson et al., 2020; Shin et al., 2018; Tedesco Triccas et al., 2019). Further research should be conducted to investigate the influence and mechanism of age on spasticity.

In our study, patients with more severe spasticity had higher NIHSS scores. These results reflect those of Shin et al. (2018), who also found that the NIHSS had the most significant correlation with the aggravation of spasticity between 3 and 12 months after stroke. Therefore, as mentioned by Opheim et al. (2015), the assessment of NIHSS scores at admission may provide a good indication of the probability of a patient developing spasticity.

Studies have shown that smoking and drinking were associated with stroke (O'Donnell et al., 2016; Yang et al., 2021), while their association with post‐stroke spasticity was unclear. Our research identified smoking history as a predictor for more severe disability from spasticity. This finding was also reported by Leathley et al. (2004). Moreover, our results revealed that a history of drinking was associated with severe spasticity. However, we could further examine the relationship between smoking/drinking and spasticity because the study data did not include a detailed smoking/drinking history. Future studies examining post‐stroke spasticity should consider smoking/drinking history.

Spasticity is a symptom of an upper motor neuron syndrome (Tedesco Triccas et al., 2019), which occurs due to a change in the net balance of excitatory and inhibitory inputs on spinal‐level circuitry secondary to injury in higher‐order centers or their descending pathways (i.e., pyramidal or parapyramidal fibers) (Li & Francisco, 2015). Little is known about the relationship between ICH lesion locations and spasticity. Cheung et al. and Lee et al. reported that the putamen, white matter tracts, and striatum influenced the development of spasticity in patients with stroke using lesion‐symptom mapping methods (Lee et al., 2019). Thus, we investigated the association between spasticity and ICH lesion sites using logistic regression. Moreover, we identified the brainstem as the lesion site with the highest incidence of spasticity. A possible explanation for this may be that the most important descending pathways of the human motor system, including the reticulospinal (RST), vestibulospinal, rubrospinal, and tectospinal pathways, originate from the brainstem (Li & Francisco, 2015). Moreover, the dorsal RST provides a powerful inhibitory effect on the spinal stretch reflex (Li et al., 2019), and abnormalities in RST outflow are considered to play a major role in the genesis of spasticity in humans (Li & Francisco, 2015). Therefore, damage to the brainstem can result in the highest incidence of spasticity. Another important finding was that the risk of moderate‐to‐severe spasticity after thalamic hemorrhage was reduced compared to that in the basal ganglia. These results may be due to imbalanced descending regulations (Li & Francisco, 2015). The corticospinal tract (CST) and corticoreticular tract are adjacent to each other in the corona radiata and internal capsule (Jang & Lee, 2019; Yeo et al., 2020); thus, damage often occurs in both the CST and corticoreticular tract, resulting in the loss of cortical facilitatory input to the medullary inhibitory center (Mukherjee & Chakravarty, 2010). Moreover, the CST is one of the most important descending pathways of the human motor system. Therefore, spastic hemiplegia is frequently observed in patients with basal ganglia ICH.

## 5 LIMITATIONS

The present study was a cross‐sectional analysis and not a longitudinal analysis; thus, it had some limitations. First, it was a single‐center study though the study has an appropriate sample size; therefore, the results may not be generalizable to all patients. Moreover, some patients with ICH may present with multiple lesions. Such cases might have attenuated the differences in spasticity observed across ICH lesion locations. Further, limited clinical information was available (e.g., detailed smoking/drinking history), so we could not further examine the relationship between smoking/drinking and spasticity. Finally, the predictors identified in this study only appear to explain some of the variability of the incidence/severity of post‐stroke spasticity, indicating other contributing factors that we did not consider.

## 6 CONCLUSION

NIHSS score is a significant predictor of post‐stroke spasticity. Logistic regression analyses revealed that lower age, higher NIHSS scores, and drinking may increase the risk of moderate‐to‐severe spasticity, while smoking and ICH in the thalamus may reduce this risk. These findings may help identify the risk of post‐stroke spasticity and prioritizing early prevention and treatment.

## AUTHOR CONTRIBUTIONS

Chang‐Yue Gao and Ling‐Yi Liao conceived the study. Ling‐Yi Liao, Pei‐Dong Xu, Xiang‐Qin Fang, Huan Cheng, and Yong Tao collected data. Qing‐Hua Wang, Pei‐Dong Xu, and Ling‐Yi Liao oversaw the statistical analysis plan, conducted the statistical analysis, and contributed to data interpretation. Xiang‐Qin Fang and Pei‐Dong Xu contributed to data quality assurance and data quality analysis. Ling‐Yi Liao drafted the initial manuscript and Chang‐Yue Gao, Pei‐Dong Xu, Xiang‐Qin Fang, Qing‐Hua Wang, Yong Tao, and Huan Cheng critically revised the manuscript. All authors gave final approval for publication.

## CONFLICT OF INTEREST STATEMENT

The authors have no conflict of interest to declare.

### PEER REVIEW

The peer review history for this article is available at https://publons.com/publon/10.1002/brb3.2906.

## Supporting information

Supplementary Material

## Data Availability

N/A

## References

[brb32906-bib-0001] Bohannon, R. W. , & Smith, M. B. (1987). Interrater reliability of a modified Ashworth scale of muscle spasticity. Physical Therapy, 67(2), 206–7. 3809245 10.1093/ptj/67.2.206

[brb32906-bib-0002] Francisco, G. E. , Balbert, A. , Bavikatte, G. , Bensmail, D. , Carda, S. , Deltombe, T. , Draulans, N. , Escaldi, S. , Gross, R. , Jacinto, J. , Ketchum, N. , Molteni, F. , Moraleda, S. , ODell, M. W. , Reebye, R. , Säterö, P. , Verduzco‐Gutierrez, M. , Walker, H. , & Wissel, J. (2021). A practical guide to optimizing the benefits of post‐stroke spasticity interventions with botulinum toxin A: An international group consensus. Journal of Rehabilitation Medicine, 53(1), jrm00134. 33057730 10.2340/16501977-2753 PMC8772370

[brb32906-bib-0003] Glaess‐Leistner, S. , Ri, S. J. , Audebert, H. J. , & Wissel, J. (2021). Early clinical predictors of post stroke spasticity. Topics in Stroke Rehabilitation, 28(7), 508–18. 33156735 10.1080/10749357.2020.1843845

[brb32906-bib-0004] Jang, S. H. , & Lee, S. J. (2019). Corticoreticular tract in the human brain: A mini review. Frontiers in Neurology, 10, 1188. 31803130 10.3389/fneur.2019.01188 PMC6868423

[brb32906-bib-0005] Jin, Y. , & Zhao, Y. (2018). Post‐stroke upper limb spasticity incidence for different cerebral infarction site. Open Med (Wars), 13, 227–31. 29876525 10.1515/med-2018-0035 PMC5984557

[brb32906-bib-0006] Katoozian, L. , Tahan, N. , Zoghi, M. , & Bakhshayesh, B. (2018). The onset and frequency of spasticity after first ever stroke. Journal of the National Medical Association, 110(6), 547–52. 30129501 10.1016/j.jnma.2018.01.008

[brb32906-bib-0007] Leathley, M. J. , Gregson, J. M. , Moore, A. P. , Smith, T. L. , Sharma, A. K. , & Watkins, C. L. (2004). Predicting spasticity after stroke in those surviving to 12 months. Clinical Rehabilitation, 18(4), 438–43. 15180128 10.1191/0269215504cr727oa

[brb32906-bib-0008] Lee, K. B. , Hong, B. Y. , Kim, J. S. , Sul, B. , Yoon, S. C. , Ji, E. K. , Baek Son, D. , Hwang, B. Y. , & Lim, S. H. (2019). Which brain lesions produce spasticity? An observational study on 45 stroke patients. PLoS One, 14(1), e0210038. 30677069 10.1371/journal.pone.0210038 PMC6345431

[brb32906-bib-0009] Li, S. (2017). Spasticity, Motor recovery, and neural plasticity after stroke. Frontiers in Neurology, 8, 120. 28421032 10.3389/fneur.2017.00120 PMC5377239

[brb32906-bib-0010] Li, S. (2020). Ankle and foot spasticity patterns in chronic stroke survivors with abnormal gait. Toxins (Basel), 12(10), 646. 33036356 10.3390/toxins12100646 PMC7600702

[brb32906-bib-0011] Li, S. , Chen, Y. T. , Francisco, G. E. , Zhou, P. , & Rymer, W. Z. (2019). A unifying pathophysiological account for post‐stroke spasticity and disordered motor control. Frontiers in Neurology, 10, 468. 31133971 10.3389/fneur.2019.00468 PMC6524557

[brb32906-bib-0012] Li, S. , & Francisco, G. E. (2015). New insights into the pathophysiology of post‐stroke spasticity. Frontiers in Human Neuroscience, 9, 192. 25914638 10.3389/fnhum.2015.00192 PMC4392691

[brb32906-bib-0013] Lundstrom, E. , Terent, A. , & Borg, J. (2008). Prevalence of disabling spasticity 1 year after first‐ever stroke. European Journal of Neurology, 15(6), 533–9. 18355307 10.1111/j.1468-1331.2008.02114.x

[brb32906-bib-0014] Mahmood, A. , Veluswamy, S. K. , Hombali, A. , Mullick, A. , N, M. , & Solomon, J. M. (2019). Effect of transcutaneous electrical nerve stimulation on spasticity in adults with stroke: A systematic review and meta‐analysis. Archives of Physical Medicine and Rehabilitation, 100(4), 751–68. 30452892 10.1016/j.apmr.2018.10.016

[brb32906-bib-0015] Meseguer‐Henarejos, A. B. , Sanchez‐Meca, J. , Lopez‐Pina, J. A. , & Carles‐Hernandez, R. (2018). Inter‐ and intra‐rater reliability of the modified Ashworth scale: A systematic review and meta‐analysis. European Journal of Physical and Rehabilitation Medicine, 54(4), 576–90. 28901119 10.23736/S1973-9087.17.04796-7

[brb32906-bib-0016] Mukherjee, A. , & Chakravarty, A. (2010). Spasticity mechanisms—For the clinician. Frontiers in Neurology, 1, 149. 21206767 10.3389/fneur.2010.00149 PMC3009478

[brb32906-bib-0017] Nam, K. E. , Lim, S. H. , Kim, J. S. , Hong, B. Y. , Jung, H. Y. , Lee, J. K. , Yoo, S. D. , Pyun, S.‐B. , Lee, K. M. , Lee, K. J. , Kim, H. , Han, E. Y. , & Lee, K. W. (2019). When does spasticity in the upper limb develop after a first stroke? A nationwide observational study on 861 stroke patients. Journal of Clinical Neuroscience, 66, 144–8. 31088768 10.1016/j.jocn.2019.04.034

[brb32906-bib-0018] O'Donnell, M. J. , Chin, S. L. , Rangarajan, S. , Xavier, D. , Liu, L. , Zhang, H. , Rao‐Melacini, P. , Zhang, X. , Pais, P. , Agapay, S. , Lopez‐Jaramillo, P. , Damasceno, A. , Langhorne, P. , McQueen, M. J. , Rosengren, A. , Dehghan, M. , Hankey, G. J. , Dans, A. L. , & Elsayed, A. (2016). Global and regional effects of potentially modifiable risk factors associated with acute stroke in 32 countries (INTERSTROKE): A case‐control study. Lancet, 388(10046), 761–75. 27431356 10.1016/S0140-6736(16)30506-2

[brb32906-bib-0019] Opheim, A. , Danielsson, A. , Alt Murphy, M. , Persson, H. C. , & Sunnerhagen, K. S. (2015). Early prediction of long‐term upper limb spasticity after stroke: Part of the SALGOT study. Neurology, 85(10), 873–80. 26276377 10.1212/WNL.0000000000001908 PMC4560058

[brb32906-bib-0020] Persson, C. U. , Holmegaard, L. , Redfors, P. , Jern, C. , Blomstrand, C. , & Jood, K. (2020). Increased muscle tone and contracture late after ischemic stroke. Brain and Behavior, 10(2), e01509. 31893564 10.1002/brb3.1509 PMC7010575

[brb32906-bib-0021] Picelli, A. , Tamburin, S. , Gajofatto, F. , Zanette, G. , Praitano, M. , Saltuari, L. , Corradini, C. , & Smania, N. (2014). Association between severe upper limb spasticity and brain lesion location in stroke patients. BioMed Research International, 2014, 162754. 24963473 10.1155/2014/162754 PMC4055577

[brb32906-bib-0022] Salazar, A. P. , Pinto, C. , Ruschel Mossi, J. V. , Figueiro, B. , Lukrafka, J. L. , & Pagnussat, A. S. (2019). Effectiveness of static stretching positioning on post‐stroke upper‐limb spasticity and mobility: Systematic review with meta‐analysis. Annals of Physical and Rehabilitation Medicine, 62(4), 274–82. 30582986 10.1016/j.rehab.2018.11.004

[brb32906-bib-0023] Schinwelski, M. J. , Sitek, E. J. , Waz, P. , & Slawek, J. W. (2019). Prevalence and predictors of post‐stroke spasticity and its impact on daily living and quality of life. Neurologia i Neurochirurgia Polska, 53(6), 449–57. 31845749 10.5603/PJNNS.a2019.0067

[brb32906-bib-0024] Shin, Y. I. , Kim, S. Y. , Lee, H. I. , Kim, D. Y. , Lee, J. , Sohn, M. K. , Lee, S. ‐G. , Oh, G. ‐J. , Lee, Y. ‐S. , Joo, M. C. , Han, E. Y. , Han, J. , Moon, M. H. , Chang, W. H. , Kim, Y. , & Kim, Y. ‐H. (2018). Association between spasticity and functional impairments during the first year after stroke in Korea: The KOSCO study. American Journal of Physical Medicine & Rehabilitation, 97(8), 557–64. 29509548 10.1097/PHM.0000000000000916

[brb32906-bib-0025] Tedesco Triccas, L. , Kennedy, N. , Smith, T. , & Pomeroy, V. (2019). Predictors of upper limb spasticity after stroke? A systematic review and meta‐analysis. Physiotherapy, 105(2), 163–73. 30745061 10.1016/j.physio.2019.01.004

[brb32906-bib-0026] Ward, A. B. (2012). A literature review of the pathophysiology and onset of post‐stroke spasticity. European Journal of Neurology, 19(1), 21–7. 21707868 10.1111/j.1468-1331.2011.03448.x

[brb32906-bib-0027] Yang, W. , Kang, D. W. , Ha, S. Y. , & Lee, S. H. (2021). Drinking patterns and risk of ischemic stroke in middle‐aged adults: Do beneficial drinking habits indeed exist? Stroke, 52(1), 164–71. 33148143 10.1161/STROKEAHA.120.032144

[brb32906-bib-0028] Yeo, S. S. , Jang, S. H. , Park, G. Y. , & Oh, S. (2020). Effects of injuries to descending motor pathways on restoration of gait in patients with pontine hemorrhage. Journal of Stroke and Cerebrovascular Diseases, 29(7), 104857. 32409256 10.1016/j.jstrokecerebrovasdis.2020.104857

[brb32906-bib-0029] Zeng, H. , Chen, J. , Guo, Y. , & Tan, S. (2020). Prevalence and risk factors for spasticity after stroke: A systematic review and meta‐analysis. Frontiers in Neurology, 11, 616097. 33551975 10.3389/fneur.2020.616097 PMC7855612

